# A review of potential microbiome-gut-brain axis mediated neurocognitive conditions in persons living with HIV

**DOI:** 10.1016/j.bbih.2020.100168

**Published:** 2020-11-02

**Authors:** Shannan Rich, Emily Klann, Vaughn Bryant, Veronica Richards, Akemi Wijayabahu, Kendall Bryant, Volker Mai, Robert Cook

**Affiliations:** a Department of Epidemiology, College of Public Health and Health Professions and College of Medicine, University of Florida, Gainesville, FL, USA; b Emerging Pathogens Institute, University of Florida, Gainesville, FL, USA; c Department of Clinical and Health Psychology, University of Florida, Gainesville, FL, USA; d Alcohol and HIV/AIDS Research, National Institute on Alcohol Abuse and Alcoholism, Bethesda, MD, USA

**Keywords:** Microbiome, Microbiome-gut-brain axis, HIV, Neurocognition, Intervention, Probiotics

## Abstract

The microbiome-gut-brain axis, or the various interactions between the gut microbiome and the brain, has been of recent interest in the context of precision medicine research for a variety of disease states. Persons living with human immunodeficiency virus (PLWH) experience higher degrees of neurocognitive decline than the general population, correlating with a disruption of the normal gut microbiome composition (i.e. dysbiosis). While the nature of this correlation remains to be determined, there is the potential that the microbiome-gut-brain axis contributes to the progression of this disease. Previous research has established that the pathology associated with HIV induces alterations in the composition of gut microbiome, including a shift from *Bacteroides* to *Prevotella* dominance, and compromises gut barrier integrity, which may promote microbial translocation and consequent systemic inflammation and exacerbation of neuroinflammation. Further, though the use of antiretroviral therapy has been found to partially counteract HIV-related dysbiosis, it may also induce its own dysbiosis patterns, presenting a unique challenge for this research.

More recent research has suggested the gut microbiome as a target for therapeutic interventions to improve symptoms associated with a variety of disease states, including HIV. Early findings are promising and warrant further research regarding the gut microbiome as a potential modifiable factor to improve health outcomes for PLWH. This review will discuss the current knowledge concerning the neuropathogenesis of HIV in the brain, role of the gut microbiome in neuroinflammation, and the relationship between HIV-status and the gut microbiome, followed by a conclusion that synthesizes this information within the context of the microbiome-gut-brain axis among PLWH. This review will also highlight the limitations of existing studies and propose future directions of this research.

## Highlights

• The microbiome-gut-brain axis has been of recent interest as a potential novel target for precision medicine.
• Individuals with HIV experience considerable neurocognitive decline and disruption of the normal gut microbiome composition.
• Probiotic supplementation has been found to reduce neuroinflammation and improve neurocognition in individuals with HIV.
• Future research controlling for confounders, using diverse samples, and evaluating long-term interventions is needed.

## 1 Introduction

**Fig. 1:**
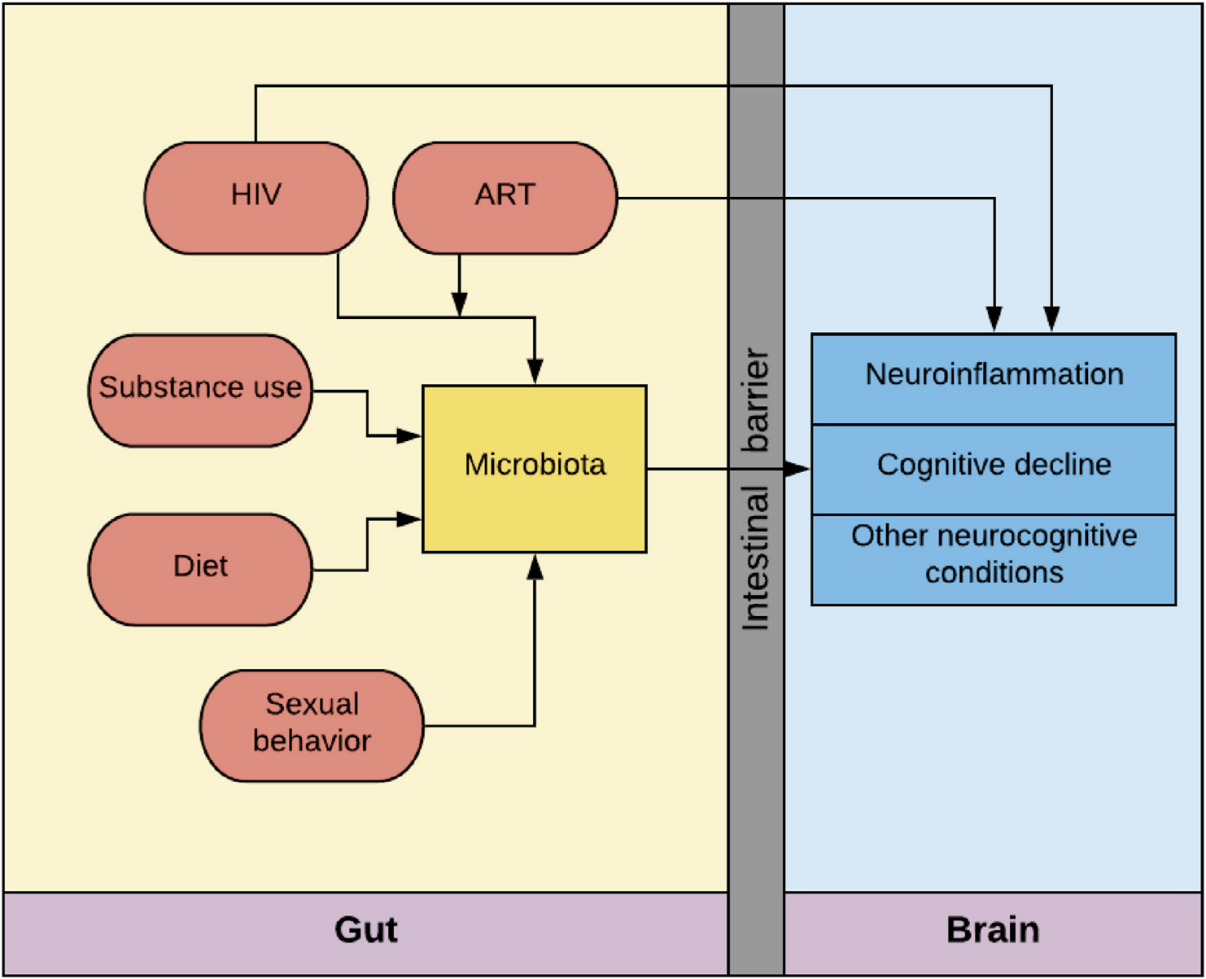
Microbiota contributions to gut-brain-axis mediated neurocognitive conditions in persons living with HIV in the presence of other modifiable risk factors. Abbreviations: HIV, human immunodeficiency virus; ART, antiretroviral therapy.

The interplay of the microbiome-gut-brain axis is an emerging area of precision medicine research. Recent research has highlighted the role of the microorganisms living on and inside of human hosts in the development and progression of several disease states, such as inflammatory bowel syndrome, autism, mental health disorders, and neurological conditions, as well as in a number of critical health functions, including for gut (Shreiner et al., 2015), liver (Tripathi et al., 2018), and brain (Zhu et al., 2020) health. Further research suggests the microbiome-gut-brain axis exists as a bidirectional and complex network of mechanisms through which a multitude of factors are hypothesized to impact brain health and cognitive functioning. Persons living with human immunodeficiency virus (PLWH), a chronic immunosuppressive infection, experience higher degrees of neurocognitive decline than the general population (Seider et al., 2014). PLWH also experience disruptions of the healthy gut microbiome (i.e. dysbiosis) (Vujkovic-Cvijin et al., 2013), which may interfere with normal microbiome-gut-brain processes. HIV-associated alterations in the gut microbiota may contribute to neurocognitive changes through the gut-brain axis. However, research which adequately controls for possible confounding factors such as HIV medications, viral suppression status, sexual behaviors, and diet, is lacking. This review will discuss the neuropathogenesis of HIV in the brain, role of the gut microbiome in neuroinflammatory processes, and relationship between HIV-status and the gut microbiome (see Fig. 1), followed by a conclusion that synthesizes this information within the context of the microbiome-gut-brain axis among PLWH. This review will also highlight notable limitations of previous studies, ultimately culminating in a recommendation for more attention to this dynamic relationship of the microbiome-gut-brain axis in the field of HIV and microbiome research.

### 1.1 HIV and the brain

The advent of combined antiretroviral therapy (ART) has vastly improved patient outcomes and revolutionized the field of human immunodeficiency virus (HIV) research. Persons living with HIV (PLWH) who achieve sustained viral suppression on ART now reach near-normal life expectancies, nearly doubling the proportion of PLWH over the age of 50 since 2015 (Mahy et al., 2014; Trickey et al., 2017). Despite improvements in life expectancy, PLWH continue to experience excess morbidity and mortality associated with age-related illnesses, including cognitive impairment and neurocognitive disorders, which demands closer attention (Grant et al., 1992; Guaraldi et al., 2011; Heaton et al., 2011; Sacktor et al., 2016; Simioni et al., 2009).

One hypothesis developed to explain this excess morbidity in neurocognitive impairment in PLWH is related to HIV neuropathogenesis. Infection with HIV can lead to neuroinflammation and premature aging of the brain, each contributing to neurocognitive decline (Guaraldi et al., 2011). HIV infection compromises the integrity of the blood-brain-barrier, a selectively permeable barrier that prevents the entry of pathogens or toxins into the brain (Atluri et al., 2015; Kamkwalala and Newhouse, 2017; Wolburg and Lippoldt, 2002). With the altered permeability of the blood-brain-barrier, HIV particles within the host CD4 adaptive immune cells can easily travel across this barrier by first infecting the brain microvascular endothelial cells and then the brain parenchyma (Atluri et al., 2015; Kamkwalala and Newhouse, 2017; Wolburg and Lippoldt, 2002). Interestingly, recent evidence indicates presence of larger pathogens, such as bacteria, in the brain tissue (Roberts et al., 2018). However, these studies were conducted exclusively on postmortem brain samples, and the exact mechanism through which HIV compromises blood-brain-barrier function to allow for microbial translocation is not fully understood. The human central nervous system (CNS) is considered to be a reservoir of HIV infection and replication as many forms of ART cannot fully penetrate the blood-brain-barrier to gain access to HIV-infected cells (Gupta et al., 2019). The penetrability of these medications through the blood-brain-barrier is variable and dependent on multiple factors including the molecular weight, method of protein binding, and lipophilicity of each compound (Ene et al., 2011). This process allows the virus to continue replicating in the brain unhindered by ART, allowing it to establish a chronic infection (Gupta et al., 2019). The presence of HIV in the CNS triggers the release of neurotoxic cytokines which contribute to persistent neuroinflammation (Kamkwalala and Newhouse, 2017). In addition to chronic neuroinflammation, HIV neuropathogenesis has also been hypothesized to occur through mechanisms of oxidative stress, metabolic disturbances, immune senescence, and gut bacterial processes (Anthony and Bell, 2008; Cañizares et al., 2014; Everall et al., 2009).

Estimates report nearly half of all PLWH suffer from HIV-associated neurocognitive decline (HAND) (Antinori et al., 2007; Chen et al., 2014; Heaton et al., 2011; Sacktor et al., 2016), which is divided into three phenotypic subtypes: HIV-associated asymptomatic neurocognitive impairment (ANI), HIV-associated mild neurocognitive disorder (MND), and HIV-associated dementia (HAD) (Antinori et al., 2007). Functional impairment levels vary by subtype – with some caveats, and range from no impairment with asymptomatic neurocognitive impairment (ANI) to mild interference (i.e. self-reported reduced mental acuity) with mild neurocognitive disorder (MND), and marked interference with daily activities with HAD (Manji et al., 2013). Multiple factors have been hypothesized to influence the likelihood of developing HAND, including age at HIV infection, duration of time spent without treatment, host genetic factors, substance use, and viral clade subtype (Kamkwalala and Newhouse, 2017; Manji et al., 2013). Since not all neurocognitive pathologies experienced by PLWH are believed to occur as a direct result of HIV infection, this review will consider a range of associated conditions, which we henceforth refer to as neurocognitive impairment.

The neuropathology associated with HIV shares many characteristics and pathways with that of Alzheimer’s disease (AD); therefore, it is currently unclear whether PLWH are truly at greater risk for AD and other similar dementias compared to the general population (Canet et al., 2018). As the population of PLWH ages, more epidemiologic research will be needed to elucidate the relationship between HIV infection and consequent dementias, apart from HAND. Long-term HIV infection has been associated with an increase in levels of amyloid beta, which, in addition to aging, is a risk factor for dementia (Rempel and Pulliam, 2005).

Another study conducted among PLWH reported that having at least one apolipoprotein E4 (ApoE4) allele, a particular ApoE genotype thought to be associated with late-onset Alzheimer’s disease (Montufar et al., 2017), was associated with decreased cognitive performance, and reduced white matter in the brain, and potentially exacerbated by existing HIV-related pathology (Wendelken et al., 2016). While accumulating evidence suggests ApoE may influence tau-mediated mechanisms of neurodegeneration (Yamazaki et al., 2019), the true role of this protein in the progression of neurocognitive impairment among PLWH is still largely undetermined. Although the rates of diagnosed dementia in this population are currently quite low, more studies are needed on the topic of age-related neurogenerative disorders in PLWH given the increasing proportion of this population entering elderly age.

In addition to having an increased burden of neurocognitive disorders and dementias, PLWH also tend to have higher levels of depression and substance use than those without HIV (Skalski et al., 2015). These mental health and substance use patterns are often linked to psychosocial factors including uncertainty, stigma, and resilience (Furlotte and Schwartz, 2017), however, some biological mechanisms have also been proposed to explain this relationship (e.g. genetic predisposition (Mutumba and Harper, 2015)). Further, a recent study involving a mouse model of alcohol-induced depression found that dietary supplementation of nicotinamide riboside (an alternative form of vitamin B3) led to increased richness and diversity of the gut microbiome as well as improvement of alcohol-induced depressive-like behaviors (Jiang et al., 2019). This example highlights the potential of the gut microbiome as a possible modifiable factor to possibly reduce the burden of mental health disorders and warrants further research among PLWH.

### 1.2 Microbiome and the brain

The human microbiome is defined as a collection of microorganisms, their genes, and their associated metabolites that occupy diverse anatomical sites both on and within the human body (Clemente et al., 2012). The microbiome, particularly that which resides in the gut or intestines, has been hypothesized to impact human brain health through a variety of mechanisms, collectively giving rise to the emerging concept of the microbiome-gut-brain axis. The proposed mechanisms underlying this relationship include (a) the simultaneous development and maturation of the microbiome, gastrointestinal tract, and hippocampal neurogenesis during early development (Ogbonnaya et al., 2015), (b) inflammation of the CNS through chronic low-level stimulation of the innate immune system by structural bacterial components (such as lipopolysaccharides [LPS]), bacterial translocation through increased intestinal permeability, and dysbiosis, (c) dysfunctional adaptive immune response due to molecular mimicry (e.g. human body reacting to bacterial antigens that mimic antigens produced by the human body), (d) transfer of gut bacterial signals between the enteric nervous system (ENS) and the brain through the vagus nerve, and (e) gut bacteria production of hormones, neurotransmitters, and metabolites that may either directly or indirectly affect the brain (Galland, 2014).

Studies of germ-free mice have enhanced the current understanding of the gut-brain axis. Germ-free mice tend to have elevated levels of serum corticosterone, a measure of hypothalamic-pituitary-adrenal (HPA) axis activation, relative to specific pathogen free (SPF) colonized controls (Sudo et al., 2004). Additionally, germ-free mice demonstrate deficits in non-spatial and working memory (Gareau et al., 2011), unique behaviors related to physical movement, social interactions, stress response, and anxiety compared to SPF mice (Diaz Heijtz et al., 2011; Warner, 2019). However, it should be noted that these results may be influenced by physiological responses related to stress and that results from germ-free models are not generalizable or translatable to non-germ-free models and should therefore be interpreted with caution. Vagus nerve afferent and efferent fibers allow for bidirectional communication between the gut and brain (Bonaz et al., 2018). The vagus nerve is a major contributor to interoceptive awareness and communicates sensitive changes in microbiota to the CNS in order to generate an appropriate response (Smith et al., 2017; Strigo and Craig, 2016). In addition to the direct gut-brain communication pathway mediated by the vagus nerve, other pathways of communication have been posited, including the endocrine pathway (HPA axis), immune (i.e. cytokine response) and metabolic (i.e. short chain fatty acids, tryptophan) (Brookes et al., 2013; Cryan and Dinan, 2012; Forsythe et al., 2016; Perez-Burgos et al., 2015; Sarkar et al., 2016).

Specific gut microbiome components have been found to be associated with specific neurodegenerative disorders, including the most common type of dementia – Alzheimer’s disease (McGettrick et al., 2018; Wang and Kasper, 2014). Mechanisms underlying this relationship are proposed to occur through neural, metabolic, endocrine, and immune pathways and include dysbiosis-induced gut permeability, secretion of amyloids and lipopolysaccharides (LPS) by gut bacteria, and generation of pro-inflammatory cytokines which contribute to Alzheimer’s pathogenesis (Giau et al., 2018; McGettrick et al., 2018; Wang and Kasper, 2014). Several murine animal model studies have been conducted to evaluate the relationship between the gut microbiome and Alzheimer’s disease states. One such study observed increased relative abundance of *Helicobacteraceae* and *Desulfovibrionaceae* and decreased relative abundance of *Prevotella* species in Alzheimer’s disease transgenic mice when compared to wild type (WT) mice (Shen et al., 2017). Numerous studies have implicated *Helicobacter pylori* infection in the pathophysiology of Alzheimer’s disease (Doulberis et al., 2018), however, a recent longitudinal, population-based study reported no association (Fani et al., 2018). Decreased relative abundance of *Prevotella* species has also been observed in Parkinson’s disease (Gerhardt and Mohajeri, 2018), and has been linked to a reduction in fecal short chain fatty acid (SCFA) concentrations. SCFAs, such as butyrate, play a role in the modulation of systemic and neural inflammation through preservation of intestinal barrier integrity (Lewis et al., 2010) and regulation of various immune cells (including neutrophils, dendritic cells, macrophages, monocytes, and T cells) (Corrêa-Oliveira et al., 2016) and are thought to be capable of direct interaction with vagus nerve afferents (Goswami et al., 2018).

Another study revealed cognitive therapeutic benefits of probiotic administration of *Bifidobacterium breve* strain A1 in transgenic mouse models of Alzheimer’s disease – reporting upregulation of the *bdnf* gene, which is involved in learning and memory processes, reduced latency time and reversed impairment of alternation behavior (Kobayashi et al., 2017). Further, a randomized controlled trial of Alzheimer’s diseases patients reported significantly higher scores on the mini-mental state examination, a widely used 30-point questionnaire to screen for cognitive impairment, after probiotic administration with *Lactobacillus* species (*L. acidophilus*, *L. casei*, and *L. fermentum*) and *Bifidobacterium bifidum* for 12 weeks (Akbari et al., 2016). Both *Lactobacillus* and *Bifidobacterium* species are known to promote the production and colonic uptake of butyrate. Additionally, decreases in plasma malondialdehyde (a biomarker of oxidative stress) and high-sensitivity C-reactive protein (a biomarker of inflammation) were observed in the probiotic supplementation group compared to the control group. Importantly, these studies reported minimal impact of probiotic administration on composition of the gut microbiome, fasting glucose levels, and lipid profiles (Akbari et al., 2016; Kobayashi et al., 2017), suggesting this intervention approach does not lead to adverse disruption of other biological processes.

In addition to contributing to neurocognitive impairment and CNS disorders, a bidirectional relationship between the gut microbiome and the brain has also been proposed to impact mental health. Gut dysbiosis patterns are associated with functional disorders of the gut and can manifest as psychological disturbances, including anxiety, depression, and substance use disorders through hormonal, humoral, and neuronal processes (Huynh et al., 2016). In a study of microbial metabolites in a murine animal model, researchers reported SCFA supplementation was effective in alleviating stress-responsiveness and improving intestinal barrier integrity in mice undergoing psychosocial stress (van de Wouw et al., 2018). Studies in humans have previously associated intestinal permeability with increased anxiety and depressed mood states, hypothesized to occur through low-grade exposure to bacterial antigens (e.g. LPS) that are capable of activating toll-like receptor 4 (TLR4), leading to initiation of signal transduction pathways responsible for innate and acquired immune response (Galland, 2014; Kelly et al., 2015). In a study conducted among adults with alcohol dependence, a bacterial species correlated with beneficial and anti-inflammatory properties, *Faecalibacterium prausnitzii*, was significantly less abundant in individuals with high intestinal permeability (Leclercq et al., 2014). Those with high intestinal permeability also had increased depression, anxiety, and alcohol craving scores after treatment compared to individuals with low intestinal permeability who recovered completely from depression and anxiety with a less severe form of alcohol dependence following treatment (Leclercq et al., 2014). This may suggest a potential correlation between gut microbiome and the physiological consequences of alcohol use disorders as well as other psychiatric disorders. There is also evidence that alcohol exposure influences gut microbial profiles, potentially mediated through changes in luminal pH (Bull-Otterson et al., 2013). A study done in rhesus macaque monkeys found chronic alcohol consumption to be associated with decreased alpha diversity (bacterial diversity and richness within a sample or sample group) and altered beta diversity (bacterial diversity and composition between samples or sample groups), observed as increased relative abundance of the phylum *Firmicutes*, of the gut microbiome compared to both baseline (pre-alcohol consumption) and controls (no alcohol consumption) (Zhang et al., 2019). Taken together, the bidirectional nature of this relationship suggests that interventions which are aimed at ameliorating disorders in one part of the microbiome-gut-brain axis (e.g. psychotherapy for depression) may impact other parts of the axis, for instance microbiome composition and functional diversity, and thus, careful attention should be given to this probable bidirectional interaction within the microbiome-gut-brain axis (Allen et al., 2017).

The mechanisms underlying the interactions between the brain and the microbiome discussed in this review thus far have focused on the indirect relationships between these entities, likely mediated through vagus nerve stimulation, immune cells, metabolites and others. However, a handful of studies have explored the potential for bacteria to colonize brain tissue (Emery et al., 2017; Roberts et al., 2018), theorizing a direct relationship between bacteria and neurological processes occurring in the CNS. A group of researchers recently revealed visual evidence of the presence of bacteria, through identification using morphological criteria, within the human brain under noninfectious conditions. More specifically, they observed bacteria, in a multitude of brain regions and cellular locations, in 34 ultrastructural samples of postmortem human brains (Roberts et al., 2018). In order to diminish the influence of possible contamination, the brains of normal and germ-free (GF) mice were fixed immediately at death and examined. Bacteria were found in similar locations within the brains of normal mice, but none were observed in GF mice, suggesting a true nonpathogenic presence of bacteria within brain tissue (Roberts et al., 2018). In a study which examined the presence of bacteria in the post-mortem brains of people with and without Alzheimer’s disease using 16S ribosomal RNA next-generation sequencing methods, researchers reported higher proportions of species of *Actinobacteria* and *Firmicutes* and lower proportions of *Proteobacteria* in the brains of Alzheimer’s patients compared to controls (Emery et al., 2017). Though the researchers could not completely rule out peri- or post-mortem microbial DNA contamination, the results of these studies warrant further investigation of both the etiology and the direct role of bacteria in the brain.

### 1.3 Microbiome-gut-brain interplay among persons living with HIV

Previous studies have demonstrated that infection with HIV induces a shift in the relative abundance of dominant bacterial taxa in the gut microbiome, from *Bacteroides* to *Prevotella* dominance, in addition to increasing the relative abundance of *Enterobacteriaceae* (Monaco et al., 2016). Increased *Enterobacteriaceae* presence is linked to a reduction in CD4 T cell counts combined with an increase in CD8 T cells, leading to chronic immune activation (Ji et al., 2018). Importantly, HIV infection is known to compromise the intestinal epithelial barrier and lead to increased intestinal permeability, possibly due to IL-18-induced epithelial cell death and decreased expression of tight junction proteins (Allam et al., 2018). Although the initiation of ART can, to some degree, improve gut epithelial damage and reverse HIV-associated gut dysbiosis, it can also lead to separate dysbiosis patterns with recently demonstrated antibacterial properties against important commensal intestinal species such as *Bacillus subtilis* and *Escherichia coli* (Shilaih et al., 2017). In a study which compared the gut microbiome of individuals with chronic (treated and untreated) versus acute HIV infection to HIV negative controls, researchers found ART was unable to completely reverse HIV-induced gut epithelial damage or fully restore HIV-associated dysbiosis (Lozupone et al., 2013). Differential effects of ART regimen type on the gut microbiome were also noted. Moreover, it has been theorized that gut microbiota can catabolize ART in vivo, as has previously been demonstrated in vitro (Klatt et al., 2017), suggesting a bidirectional association between ART use and gut microbiome composition (Crakes and Jiang, 2019; Li et al., 2016; Pinto-Cardoso et al., 2018). In fact, a recent study has shown that vaginal microbiota, most profoundly *G. vaginalis* and other anaerobic bacteria, are capable of metabolizing and therefore depleting tenofovir (topical ART gel commonly used for pre-exposure prophylaxis) in vivo (Klatt et al., 2017). However, the effect of the microbiota within the gut on ART metabolism and efficacy in PLWH remains largely unknown.

One hypothesis for the persistent neuroinflammation experienced by PLWH implicates the gut microbiome as one potential source. Higher levels of *Bacteroides* and lower levels of *Firmicutes* in the gut has previously been associated with HAD – the most severe form of HAND (Perez-Santiago et al., 2017). Mechanisms proposed to explain this theory include the contribution of gut microbiome-induced elevation in LPS levels and consequent immune activation to neurocognitive dysfunction in this population (Ancuta et al., 2008; Lyons et al., 2011). In a study conducted among neuro-asymptomatic PLWH on ART, markers of neuroinflammation and white matter abnormalities were associated with microbial translocation (Vera et al., 2015). Further, CD14, an innate immunity marker of monocyte activation and microbial translocation, has been associated with impaired neurocognitive functioning (Lyons et al., 2011). Levels of this molecule can be measured in plasma, and it has been recommended as a potential biomarker for monitoring HAND progression in PLWH (Lyons et al., 2011).

Similar to the idea that bacteria may have a direct role in colonizing the brain and causing neurological damage or disorders such as Alzheimer’s, some researchers have been interested in exploring the contribution of microbial populations in the brain to health outcomes of PLWH. Despite this interest, the literature published on this topic is sparse. In one study which employed deep sequencing methods to assess microbial presence and diversity in the postmortem brains of PLWH and non-diseased controls, researchers reported high relative abundance of *Proteobacteria* and no presence of *Firmicutes* organisms in the brain samples, despite these organisms being commonly present in most other microbial communities at other human body sites (Branton et al., 2013). However, these findings were similar across the study population (in both persons with and without HIV), and the researchers did not report any clear distinctions between the populations with respect to microbial population composition.

Perhaps the most compelling evidence of the microbiome-gut-brain axis among PLWH has been derived from intervention studies in which researchers intentionally modified the gut microbiome to observe impacts on brain health. Two studies to date have examined the effects of probiotic supplementation among PLWH and both reported yielding significant neurological benefit, including reduced neuroinflammation and neurocognitive impairment (Ceccarelli et al., 2017a, 2017b), further suggesting the importance of the gut microbiome on neurocognition. In one study of 35 PLWH, those with the highest levels of neuroinflammation (defined as the highest levels of neopterin – a biomarker of monocyte activation in cerebrospinal fluid) were assigned to the intervention arm (N=9) and received the oral probiotic supplement containing the following species: “*Lactobacillus plantarum* DSM 24730, *Streptococcus thermophilus* DSM 24731, *Bifidobacterium breve* DSM 24732, *Lactobacillus paracasei* DSM 24733, *Lactobacillus delbrueckii subsp. bulgaricus* DSM 24734, *Lactobacillus acidophilus* DSM 24735, *Bifidobacterium longum* DSM 24736, and *Bifidobacterium infantis* DSM 24737” (Ceccarelli et al., 2017a). After the six-month probiotic treatment intervention, researchers observed significantly decreased levels of neopterin among participants in the intervention group, but not the control group, compared to their levels at baseline. Further, the researchers also observed improvement in the intervention arm for nearly all neurocognitive tests that were performed, including the auditory verbal learning tests of immediate and delayed recall, and the semantic, phonological, and verbal fluency tests, among others (Ceccarelli et al., 2017a). The findings were only statistically significant for tests of immediate recall, delayed recall, and verbal fluency, however, which is likely due to the small size of the study population. In another study published by the same group, researchers observed significant improvement in neuropsychological test performance as well as decreased levels of cerebrospinal fluid microRNA-29a-c levels in PLWH, who received the same probiotic supplementation mentioned previously, compared to controls (Ceccarelli et al., 2017b). MicroRNAs are believed to modulate HIV infection and can serve as a biomarker for confirming undetectable levels of virus (Ceccarelli et al., 2017b). Future studies in more diverse populations and geographic regions are warranted in order to assess the validity, reliability, and generalizability of these promising results.

### 1.4 Limitations of current research

Previous studies on the relationship between the microbiome and neurocognitive dysfunction among PLWH have numerous limitations, which limit the interpretability of the results in the context of therapeutic intervention development. These studies tended to be conducted in predominantly younger populations (under 50 years old) who often suffer fewer neurocognitive dysfunctions and metabolic disturbances, calling into question the representativeness of the sample populations and the generalizability of the results to the general target population of PLWH. Additionally, many microbiome-disease association studies in PLWH fail to control for factors that influence the microbiome separately from HIV infection, including ART use and adherence, dietary habits (including use of probiotics), sexual behaviors, substance use, long-term exposure to certain medications, physical activity, and other comorbidities. For example, one study reported no evidence of an association between HIV infection and gut microbiome dysbiosis after controlling for sexual orientation (Noguera-Julian et al., 2016).

However, in another study conducted among PLWH, anal sex and substance use were among the most significant predictors of microbiome variation (Fulcher et al., 2018). In their permutational multivariate analysis of variation (PERMANOVA), researchers observed multiple associations between self-reported sexual practices, including oral and receptive anal sex, and the presence of certain bacterial genera in the gut microbiome (Fulcher et al., 2018). Oral sex was associated with increased presence of *Granulicatella* and *Clostridium* cluster XIVa species and decreased presence of *Actinomyces*, *Campylobacter*, and *Firmicutes* classes whereas, receptive anal sex practices were associated with increased presence of *Peptostreptococcus* and *Anaerococcus* species, and likewise decreased presence of *Firmicutes* organisms (Fulcher et al., 2018). Different relative abundances of bacterial genera were also reported on the basis of recency and frequency of sex practices and the presence of coinfection with other sexually transmitted diseases (Fulcher et al., 2018). Further, another study observed significantly higher alpha diversity of the gut microbiome in men who have sex with men (MSM) compared to men who have sex with women (MSW), regardless of HIV status (Armstrong et al., 2018). Marijuana use was associated with increased relative abundance of *Solobacterium, Ruminococcus*, *Clostridium cluster IV*, and *Fusobacterium* species and decreased abundance of *Prevotella, Acidaminococcus*, *Anaerostipes*, *Dialister*, and *Dorea* species (Fulcher et al., 2018). The increased abundance of these organisms has been hypothesized to increase inflammation and gut permeability through arachidonyl-ethanolamide and cannabinoid receptor 1 agonism, or conversely decrease inflammation through production of arachidonoyl-glycerol (Fulcher et al., 2018). Additionally, methamphetamine use was associated with an increased relative abundance of *Porphyromonas* and *Granulicatella* species and a decreased relative abundance of *Collinsella, Ruminococcus*, and *Parabacteroides* species (Fulcher et al., 2018). Some *Porphyromonas* species are believed to promote systemic inflammatory cytokines (Arimatsu et al., 2015), which may explain the increased inflammatory states observed with HIV infection and chronic substance use.

Substance use has also been shown to differentially affect the risk of HAND on the basis of age, with younger persons who have a history of substance use reportedly at greater risk (Fogel et al., 2015). However, it should be noted that neurocognitive impairment consequent solely of substance use does not fit within the classification of HAND, but rather substance/medication-induced neurocognitive disorder (Sachdeva et al., 2016). Further, as the percentage of PLWH who are virally suppressed continues to increase over time, from 32% in 1997 to 86% in 2015 (Nance et al., 2018), other additional factors that commonly contribute to neurocognitive decline (e.g. substance use, medical comorbidities, etc.) are also likely applicable to this population. In studies of ethanol exposure in murine animal models, patterns of increased inflammation, intestinal barrier damage, and subsequent intestinal permeability were linked to ethanol treatment (Shao et al., 2018), suggesting that alcohol consumption in humans may lead to increased bacterial translocation, inflammation, and dysbiosis patterns. In another murine HIV mouse model, alcohol-induced dysbiosis and weakened gut barrier integrity led to compromised host immune defense against infection, irrespective of HIV status (Samuelson et al., 2019). Among PLWH, alcohol use is known to contribute to poor gut health, even among those who are virally suppressed. Additionally, the distinction between virally suppressed PLWH and those with poorly controlled HIV in studies of the microbiome-gut-brain axis has not been explicitly considered.

Given the variety of factors that may separately impact the microbiome in PLWH, future studies should seek to control for confounding variables when examining the potential mechanisms underlying the microbiome-gut-brain axis. Although potential confounders of the relationship between the microbiome-gut-brain axis and neurocognition may be difficult to adequately control for due to their frequency in PLWH, this does not diminish the necessity of research aimed at potential novel interventions for this population. Future research is needed to elucidate the underlying mechanisms of the relationship between the microbiome and neurocognitive manifestations in PLWH – specifically whether certain microbiome signatures characteristic of this population drive symptom emergence and severity or conversely are a product of the disease-state. This clarification is essential for the future development of effective precision medicine interventions.

## 2 Concluding remarks and future perspectives

In conclusion, the microbiome-gut-brain axis is a dynamic, bidirectional, and complex network of mechanisms through which a multitude of factors are hypothesized to impact overall brain health. This review highlights key areas of research in these domains, which may be particularly important to examine within the context of HIV infection. The separate proposed mechanisms of HIV- and microbiome-induced neuropathogenesis and neuroinflammation processes, and the key limitations of existing studies in these areas, were discussed. To further elucidate the relationship between the brain, gut, and microbiome in the context of reducing excess neurocognitive morbidity among PLWH, the following directions for future research in this domain are proposed.

### 2.1 Biomarker research

Determining microbial signatures of HAND, as well as other neurocognitive pathologies, will be important for identifying PLWH at greatest risk for neurocognitive decline, while also providing a potential modifiable mechanism through which their risk can be reduced. Additionally, studies which expand microbiome specimen types should also be implemented. Most studies discussed in this review concentrated on fecal samples to study the profiles of intestinal bacteria. Nevertheless, there are distinct microbial communities throughout the body and studies which consider additional specimen types, including saliva, vaginal swabs, and brain tissue, are warranted in order to characterize a more complete depiction of the microbiome in PLWH. For instance, the oral microbiome has been increasingly linked to numerous local and systemic disease states (Sampaio-Maia et al., 2016), and saliva sampling tends to be preferred over stool collection among research participants (Osborne et al., 2018). However, the role of the oral microbiome in brain health, or in the context of HIV infection, remains to be investigated. The use of diverse specimen types may promote the exploration of lesser known areas of microbiome research in the context of HIV and brain health. One area of research described in this review which warrants further attention is the role of bacterial colonization in the brains of PLWH. Future studies may consider whether infection with the virus, which maintains a reservoir in the human brain, mediates or modulates the microbial activity of the brain (and vice versa), and determine how this might influence the subsequent risk for neurological impairment.

### 2.2 Causality and the microbiome-gut-brain axis in the context of HIV

It is important to note that the microbiome studies discussed in this review do not make causal conclusions. While many associations between characteristics of the microbiome and markers of health outcomes have been established, the underlying molecular mechanisms of many of these associations remain unknown. Therefore, the nature of this association, and whether it is truly causal, also remains to be determined. For example, the shift from *Bacteroides* to *Prevotella* gut microbiome dominance observed following infection with HIV may truly influence aspects of disease severity (i.e. inflammation and neurocognitive decline), or, may simply be a product of HIV-associated physiological alterations in the gastrointestinal tract of the host. Understanding of the causal molecular mechanisms, such as bacterial metabolite production or immune modulation, underlying associations between the microbiome and target health outcomes is essential for the future development of therapeutic interventions intending to modify the gut microbiome.

### 2.3 Assessment of diet and behavior

As noted extensively in this review, lack of consideration of individual characteristics such as ART adherence, physical activity, other medication or supplement use, diet, substance use, sexual behaviors, and presence of other comorbidities in analyses of the microbiome-gut-brain axis is common and presents a concern with respect to the scientific validity of the presented conclusions. Lack of controlling for these potential confounders may obscure the true nature of the relationship between the microbiome and the brain in the context of HIV; however, the number of participants and level of resources needed to account for these factors makes this approach challenging. Aside from the analytical flaws, ignoring the social and behavioral factors, which may be independently perpetuating gut dysbiosis, also calls into question the sustainability of current intervention approaches. These approaches, which are designed to modify the gut microbiome in the short-term through prebiotic or probiotic supplementation, neglect to consider the effectiveness of these interventions in the long-term, which are dependent upon adherence, especially in the absence of any behavioral changes. Future longitudinal studies which incorporate survey data on behavioral factors, such as medication adherence, diet, sexual practices, and substance use, are necessary to determine the success of current intervention approaches. Consideration for long-term intervention is of great importance given the dynamic nature of the microbiome over time and across the human lifespan (Aleman and Valenzano, 2019).

### 2.4 Intervention studies

As mentioned previously, the potential cognitive benefits of altering the gut microbiome via dietary or behavioral modification and prebiotic or probiotic supplementation warrant additional intervention studies in this domain, with important considerations. In addition to supplementation, alternative approaches to improving gut health should be considered in this population. For example, findings from a focus group study conducted among PLWH indicated that participants did not have knowledge about the role of diet in reducing inflammation and promoting a healthy gut microbiome (Vance et al., 2017). This suggests that an area for future intervention could be to promote education concerning the ways in which PLWH can improve gut health, possibly by adhering to an anti-inflammatory diet, thereby indirectly improving brain health and cognition. In summary, neurocognitive impairment remains a significant burden for many PLWH and additional investigation of the biological pathways and intervention opportunities along the microbiome-gut-brain axis in this population is needed.
